# Addressing Test Anxiety in High-Achieving Schools: A Research-Based Approach

**DOI:** 10.3390/bs16050828

**Published:** 2026-05-21

**Authors:** Benjamin J. Lovett, Sybille Bruun-Moss, Cathy Urinyi

**Affiliations:** 1 Teachers College, Columbia University, New York, NY 10027, USA; cau2111@tc.columbia.edu; 2 Elisabeth Morrow School, Englewood, NJ 07631, USA

**Keywords:** test anxiety, high-achieving schools, academic pressure

## Abstract

Schools with very high academic achievement levels are often beset with high levels of pressure and anxiety around academics and testing in particular. In recent years, research has investigated the mechanisms that make attending high-achieving schools a risk factor for anxiety and related problems. The resulting understanding can inform appropriate strategies for addressing test anxiety. In the present paper, we review relevant research on the features of high-achieving schools that promote anxiety, and present well-established empirical facts about the nature of test anxiety, including its relationships with avoidance behaviors and with test performance. We then discuss how test anxiety manifests at high-achieving schools and present a model of how to apply evidence-based intervention strategies to address test anxiety in high-achieving settings.

## 1. Introduction

Students’ anxiety surrounding tests and other forms of evaluation at school has long been a topic for scholarly discussion. The first empirical examination of test anxiety was published over a century ago (Folin et al., 1914), where researchers found that after taking an important test, the level of sugar in some medical students’ urine rose. (This is now known to be a consequence of stress-related adrenaline release.) It is interesting that this early study examined test anxiety in a sample of high-achievers (i.e., students at Harvard Medical School). Although low academic achievement is a risk factor for some kinds of emotional concerns (e.g., Agnafors et al., 2021), test anxiety is often present in high-achieving settings, where the pressures are greater, and where students often feel that even objectively good performance is not quite good enough.

In the present paper, we offer a perspective on test anxiety in high-achieving K-12 schools (defined in greater detail in Section 2). We write as researchers but also as practitioners who work in these schools. We believe that students at high-achieving schools could benefit from research-based intervention approaches, and ones that are tailored to the unique pressures of their setting. Here, we review the research on those pressures, discuss the consensus on the “science of test anxiety,” and present a research-based approach to addressing test anxiety in high-achieving settings. Although there is much relevant research literature, it is scattered across different subfields of psychology and education, and we were unable to find any papers specifically integrating relevant literature on this issue. We conducted a narrative review of the literature on several related points: the psychological stressors present in high-achieving schools; the nature of test anxiety; the presentation of test anxiety in high-achieving schools; and management strategies for test anxiety.

We write at a time when the well-being of youth is under close examination. Mental health problems among children and adolescents have reached “crisis” levels in the United States (Office of the Surgeon General, 2021). National data indicate that there has been a substantial increase in poor mental health among children and adolescents since 2013, with 40% of high school students in the U.S. in 2023 reporting persistent feelings of sadness or hopelessness (Centers for Disease Control and Prevention [CDC], 2024, p. 55). Anxiety disorders remain among the most common mental health conditions globally as well, with notable increases following the COVID-19 pandemic (Bie et al., 2024). National data further indicate that clinically diagnosed anxiety in U.S. adolescents increased by approximately 61% between 2016 and 2023, with overall mental and behavioral health conditions rising by 35% over the same period (Sappenfield et al., 2024). The present paper focuses on the U.S. context, while in some other countries, much of the literature focuses on pressure to attend instruction and tutoring outside of the principal school where the student is enrolled (e.g., Joung & Morgan, 2025). Therefore, we are cautious in generalizing beyond the U.S. context.

## 2. The Pressures of High-Achieving Schools

Much of the scholarly literature on child mental health has focused on youth experiencing poverty, and on schools described as “high-need” or “low-resource,” where academic achievement levels tend to be lower (e.g., Galagali & Brooks, 2020; Jabbour & Pillay, 2025). This focus is appropriate and understandable, but the available data suggest that there are unique risks to being on the opposite end of these continua of wealth and achievement as well. The late Suniya Luthar, a prolific researcher, was initially surprised when she found that affluent adolescents reported higher levels of mental health problems than much poorer peers (see, e.g., Luthar, 2003). She acknowledged that “for those concerned about the next meal, the misery borne of ennui can seem ludicrous” (Luthar, 2003, p. 1590), but with some trepidation, she called on researchers to study the unique challenges experienced by youth who seem privileged (at least from the outside). Initial research in this area focused on the wealth of students’ families (e.g., Luthar & Latendresse, 2005), but over time, the studied population changed to students in high-achieving *schools*, with evidence suggesting that school-level socioeconomic composition may influence developmental outcomes above and beyond family characteristics (Coley et al., 2018). Therefore, a shift in definition was made in response to evidence suggesting that the concentration of high-achieving, affluent peers within a school environment contributes to increased pressure and risk in high-achieving schools (Luthar & Kumar, 2018). High-achieving schools are, generally speaking, defined as competitive public or private schools characterized by high admission rates to selective colleges, above-average standardized test scores, robust extracurricular programming, and parental emphasis on achievement (Luthar & Kumar, 2018; National Center for Education Statistics [NCES], 2024; D. C. Pope, 2001).

Research supports the presence of several mechanisms that place students at high-achieving schools at elevated risk for mental health problems (Demerath et al., 2010; Ebbert et al., 2019; Luthar et al., 2020). First, students within the school are often directly competing for outcomes, such as winning admission to a particular top college or receiving the school’s sole recommendation for a prestigious award. This creates a “survival of the fittest” mentality and lowers peer support that might otherwise be available. Peers become competitors rather than confidants. Second, high-achieving schools tend to have more opportunities—advanced classes, extracurricular options, special projects—but having all of these opportunities creates pressure to take advantage of them, leading to overburdened schedules and burnout. Finally, students at high-achieving schools tend to have high-achieving models to observe: parents, peers, and school professionals who are themselves living imbalanced lives and at risk for burnout but who also serve as standards that the student feels pressure to live up to.

Although our focus is on school settings, parenting deserves further comment. Parents of students at high-achieving schools do not merely serve as role models; their behavior can also raise students’ anxiety levels by promoting specific contingent self-worth beliefs. Crocker (2002) had noted that people’s self-esteem is based on success or failure in particular domains of performance; those domains become the “contingencies of self-worth.” Not all domains of contingency are equally healthy; specifically, making one’s self-worth contingent on factors that are not entirely under one’s own control predicts the development of mental health problems (Crocker & Park, 2011). Unfortunately, academic success is one of those factors, particularly at high-achieving schools that boast the most rigorous coursework. A student taking several honors and advanced placement classes may well be unable to achieve top grades in every class. In these settings, students are encouraged to excel academically while simultaneously maintaining involvement in extracurricular activities, often with the implicit understanding that these achievements are necessary for life success. As parental investment increases, so does the perceived cost of failure. Students often internalize the belief that only their high performance can justify the considerable time, effort, and resources that parents devote toward their success (Levine, 2006). With academic success highly dependent on test performance, test anxiety is an understandable response to this situation.

## 3. The Nature of Test Anxiety

Test anxiety has been defined as “the set of phenomenological, physiological, and behavioral responses that accompany concern about possible negative consequences or failure on an exam or similar evaluative situation” (Zeidner, 1998, p. 17). As that definition suggests, the experiences of test anxiety include physiological symptoms (e.g., a racing heart, a churning stomach), cognitive symptoms (e.g., worry-thoughts about an upcoming test, negative thoughts about oneself that occur during a test), and associated behaviors (e.g., procrastinating on studying for a test, or turning in a test without checking one’s work, to escape the situation). Studies have found test anxiety to be a very common experience (for review, see Jordan & Lovett, 2025), and indeed, in a large sample of college students, Lovett et al. (2024) found that 99% of students reported experiencing at least one symptom of test anxiety at least some of the time. Similarly, in other studies, most students in grades 3 to 5 reported moderate or high levels of anxiety about tests (Segool et al., 2013), and in grades 9 and 10, the average self-report rating of test anxiety suggested that students experienced the problem either “sometimes” or “often” (D. W. Putwain, 2007).

The physiological symptoms of test anxiety have been confirmed with objective physical measures (Roos et al., 2021), and painstaking thought-recording studies have done the same thing for the cognitive symptoms (see Galassi et al., 1981). Less work has been done on the behaviors associated with test anxiety, but procrastination (a common associated behavior) has been found to serve as a kind of short-term mood repair, since delaying studying avoids discomfort associated with the task (Sirois & Pychyl, 2013). Moreover, the psychological foundation of the behaviors associated with test anxiety appears to be a tendency towards avoidance (Jordan & Lovett, 2025). This tendency relates to a long-studied topic in educational psychology: students’ achievement orientations. Elliot and Church (1997) introduced two distinct motivational orientations regarding achievement: approach-oriented and avoidance-oriented motivation. Students who perceive tasks as a means to obtain a positive or desirable outcome, such as studying to achieve competence in a subject area or to demonstrate greater competence relative to their peers, possess approach motivation. Unlike approach-oriented motivation, avoidance-oriented motivation is rooted in fear of failure and concern about negative evaluation. Students endorsing avoidance goals are motivated to avoid appearing incompetent and report higher levels of test anxiety and more disorganized studying (Elliot & Church, 1997; Elliot & McGregor, 2001). Elliot and Church (1997) found that avoidance motivation was associated with lower exam performance, in part because of students limiting their opportunities to achieve mastery in their academic work.

The *cycle of avoidance*, also known as the anxiety-avoidance cycle, is a self-reinforcing pattern in which anxiety about a threatening situation leads an individual to avoid it, which provides temporary relief from symptoms but strengthens the anxiety in the long run. *Experiential avoidance*, or the attempt to avoid unpleasant thoughts, feelings, and other distressing internal experiences, plays a direct role in the cycle and has been linked with the fear of negative evaluation and even specifically with procrastination (Hayes-Skelton & Eustis, 2020; Jeffords et al., 2018). Students whose motivation is avoidance-oriented often associate studying with the anticipation of failure. Negative reinforcement then drives the cycle of avoidance, since avoiding the task reduces current discomfort (Ye et al., 2025). This short-term reduction in distress reinforces the avoidance behavior, increasing the probability that similar avoidance strategies will be used in future studying situations. As studying becomes increasingly associated with anxiety, it can become more difficult to start. Students may rely more heavily on self-protective strategies, such as procrastination, surface-level review, or avoidance of challenging material. At a larger level, avoidance can influence student choices around classes, college majors, and even careers; it is not unusual for a student to alter these choices to avoid tests (e.g., Costello et al., 2025). In more severe cases at the K-12 education level, students avoid school entirely, refusing to attend. Of course, school refusal behavior tends to make avoidance worse over time, since as the student continues to miss more work and fall farther behind in skill development, the idea of returning to school becomes more and more distressing.

Much of the research on test anxiety has involved understanding the nature of the relationship between test anxiety and test performance. In general, test anxiety is associated with modestly lower scores on cognitive and academic tests (the correlation is about *r* = −0.20; see Von der Embse et al., 2018, for a review). There are two broad classes of theories explaining that relationship (Sommer & Arendasy, 2015). Interference theories claim that anxiety directly interferes with cognitive performance during tests, preventing students from working on the test, recalling information, and the like. In essence, interference theories claim that the anxiety-performance relationship is direct and causal. In contrast, deficit theories claim that the relationship between anxiety and performance is artifactual, an incidental consequence of the fact that students with higher test anxiety tend (on average) to have weaker academic and test-taking skills. Those weaker skills lead to both (a) higher anxiety and (b) lower test performance.

Generally speaking, research has supported the deficit theories more than the interference theories. For instance, research has found that the relationship between test anxiety and test performance goes away when controlling for measures of students’ knowledge in low-stakes settings (e.g., Theobald et al., 2022). In addition, students do not generally do worse on high-stakes tests than on lower-stakes practice tests (Schlosser et al., 2019), even though the former induce more anxiety. Finally, students’ anxiety level *after* a test is a better predictor of their test performance than their anxiety level *before* a test (Zeidner, 1991), which suggests that anxiety does not actually cause lower performance.

## 4. Test Anxiety at High-Achieving Schools

Students in high-pressure achievement contexts must focus on their performance on objective indicators of achievement, and the most prominent and anxiety-inducing achievement measures are tests. Testing has long been identified as a major source of anxiety for students (D. Putwain & Daly, 2014; D. Pope & Simon, 2005), and in high-achieving schools, testing is frequent and consequential. Particularly for affluent youth, high-stakes assessment starts very early and remains a constant throughout the child’s educational journey. For these youth, the competitive nature of educational testing starts at an age and continues at a pace that is almost unimaginable for most of the general population. It appears that the greater frequency and higher stakes of testing in youth attending high-achieving schools contribute to higher levels of test anxiety.

Among this group, children as young as 18 months are evaluated as they vie for a selective spot in a top private preschool program (Diaz & Rivera, 2025; Ramey & Ramey, 2009), and many families engage in early academic preparation for school entry (Kobakhidze & Hui, 2024). In Kindergarten, children are assessed and their progress monitored for emergent literacy and math abilities (Clemens et al., 2023). By 3rd grade, many schools are administering standardized tests annually and sharing data with families who then choose to tutor students who demonstrate even mild “deficits.” In 6th grade, students are often juggling 2–3 classroom tests weekly, on top of homework and extracurricular activities, access to which is often contingent on the students’ continued successful academic performance.

Students in high-achieving middle schools are often expected to maintain strong academic records to remain competitive in the high school placement process. In many cases, students prepare for high-stakes high school admission examinations. For instance, in New York City, a local test, the Specialized High Schools Admissions Test (SHSAT), is used to compete for admission to any of eight high-performing public high schools. Nationally, students applying for admission to private high schools often take the Independent School Entrance Exam (ISEE). These assessments are characterized by their perceived rigor as well as the amount of preparation families are willing to invest to ensure higher scores by tutoring, retakes, or both. (Students can retake the ISEE once per testing season for a maximum of three retakes in a 12-month admissions cycle.) By 8th grade, excelling on assessments like the SHSAT and/or the ISEE is perceived to be the key not only to admission at the right high school, but (indirectly) to admission to a coveted spot at an elite college. Paired with the family dynamics mentioned earlier, this view of test performance creates conditions ripe for anxiety. It is no wonder that students who may not ordinarily be at risk for anxiety begin to perceive danger in the assessments they face.

## 5. Test Anxiety Management Strategies

Evidence-based strategies for managing test anxiety can be divided into (a) psychotherapy strategies for reducing anxiety symptoms, (b) skills-based approaches for studying and test-taking, and (c) school-level responses. We briefly discuss each of these below. The strategies are built specifically on the research covered above, showing that test anxiety relates to avoidance behavior, and these strategies aim to overcome that avoidance.

### 5.1. Psychotherapy Strategies

Schools often offer psychotherapy-oriented techniques through professionals such as school counselors, school psychologists, and school social workers. Although it is unusual to offer long-term therapy in school settings, short-term counseling is commonly available, and in the case of test anxiety, it is particularly appropriate to use school-based treatment, since the mental health issue that the student is experiencing relates to school achievement.

Effective psychotherapy for anxiety incorporates exposure activities, where clients directly confront the stimuli that have been causing anxiety (Kendall, 2025). Implementing this in cases of test anxiety is a bit unusual, since test-anxious students have often been exposed to tests without much benefit. Instead, in such cases, students benefit from exposure to the symptoms of anxiety in a safe place where no threat (i.e., no test) is present (Jordan & Lovett, 2025). For students who experience intense physiological panic symptoms during (or even before) tests, it is often helpful to practice interoceptive exposure exercises such as hyperventilation, to deliberately induce those symptoms (e.g., a rapid heart rate) in an environment that the student knows to be safe. Students can practice these exercises daily at home and learn that the symptoms are not dangerous; with enough practice, the symptoms are not even distracting. Cognitive symptoms often respond to a worry-journal exercise, where students practice exposing themselves to worry thoughts but in a more organized and productive manner.

Exposure-based techniques are strongly supported as core activities in psychotherapy for anxious youth, even relative to other cognitive-behavioral techniques (Kendall, 2025). However, in school settings, exposure therapies are often not used, and survey data suggest that many practitioners lack sufficient time and training to implement the techniques well (Weiss et al., 2024). We encourage practitioners to use exposure-based techniques after educating parents and students about their benefits, and after explaining to administrators that although the techniques take time to use and require proper training, they can be cost-saving in the long run due to their efficacy.

High-achieving schools often have significant resources for promoting students’ mental health, but may not always be using the most evidence-based procedures for symptom reduction. Although general supportive counseling can be helpful in some cases of anxiety (Kendall, 2025), it is far less effective than exposure-based procedures for reducing specific symptoms. Worse still, as Dunning et al. (2025) noted, there are many techniques that can actually worsen anxiety over time (even improperly implemented exposure can do this), and schools should be careful to ensure that high-quality services are being offered.

### 5.2. Training in Study and Test-Taking Skills

For students who experience high anxiety but nonetheless do well on tests, the psychotherapy procedures described earlier are often sufficient. But students who also have poor test performance and who exhibit avoidance behaviors (e.g., procrastination) in test preparation often benefit from instruction in specific science-based techniques for studying. Cognitive science and learning theory have provided reliable advice for effective studying (for a review, see Dunlosky et al., 2013). Unfortunately, many students are never given that advice, and in particular, students who have high levels of anxiety are less likely to study in efficient, effective ways (Jenifer et al., 2022).

Optimally, studying mirrors test-taking. Students should focus on self-testing, then, as the core of effective studying. Some teachers make this relatively easy by providing study guides in the form of questions that are in a similar format and cover the content that is on the exam, but often, students must create their own sample questions or other stimuli (e.g., flash cards) to self-test. The key is for students to force themselves to retrieve and apply class knowledge without access to anything (a book, notes) during the exam. This type of retrieval practice leads to lasting knowledge (Agarwal et al., 2021). Even when students have good study materials, they may use them improperly. For instance, an anxious student is particularly liable to turn over a flash card (or click an electronic one) before forcing themselves to state what is on the other side. Anxious students may also avoid studying the hardest material because it is more aversive. Retrieval practice—with the hardest material and without access to a book or notes—is doubly appropriate for anxious students, as the technique not only builds knowledge but also functions as exposure.

Test-taking skills are a related but distinct set of competencies (from study skills). These include reading directions carefully, looking through an entire exam as soon as it is handed out (so as to develop a time management strategy), checking work if there is time available, making educated guesses on selected-response items, and the like. Again, a subset of test-anxious students (usually those with lower test scores) benefit from instruction in these skills (Banks & Eaton, 2014).

High-achieving schools often have learning support resources. In public schools, the relevant supports are sometimes called “academic intervention services,” and sometimes study skills and test-taking skills can be covered in “advisory” periods as well. In private schools, learning specialists often meet one-on-one or in small groups with students who are having trouble performing on tests and then teach these skills (Vantine, 2016).

### 5.3. School-Level Responses

By “school-level responses,” we refer to strategies that apply to the student body at large, rather than working with identified students who have high levels of test anxiety. Many preventive approaches occur at the school level. One such approach involves psychoeducation—teaching students about the science of test anxiety. In particular, students often believe that test anxiety has a direct, negative effect on test performance, whereas the available evidence suggests that most students can still complete a test fairly well under conditions of anxiety, and anxiety’s effects generally happen earlier, when students are preparing for tests (e.g., Theobald et al., 2022). Students should know this so that they are less likely to shut down and give up during a test if they are experiencing significant distress. Too often, students use their anxiety symptoms as information about how they are performing, when in fact test anxiety symptoms are a poor predictor of actual test performance (Von der Embse et al., 2018).

Another school-level response involves training teachers in effective assessment practices. For instance, teachers often have minimal training in test design, and poor test design is a major cause of test anxiety. Teachers often have little idea of how much material to put on a test, may unintentionally use terms or phrasings that were not used when teaching the information, and may accidentally include topics or require skills that have not been taught. Even careless typos and unclear writing heighten test anxiety and reduce test validity. A related topic for teacher training involves letting teachers know about what types of test preparation material are appropriate to provide students with, including study advice (tailored to each teacher’s classes) and any study guides or sample questions/problems.

High-achieving schools are known for having high-level professional development events, and these events are a perfect time to cover this kind of material through hands-on, interactive workshops. We find many teachers at these schools to be genuinely intellectually interested in the science of test anxiety and in how to better support the anxious students in their classes.

To be clear, school-level responses complement rather than compete with student-level responses (such as psychotherapy and training in study skills). The two levels of responses can be viewed as fitting into different places in a service delivery model now commonly referred to as a Multi-Tiered System of Support (MTSS; see Jordan & Lovett, 2025). Tier 1, which encompasses universal strategies for all students, is a place for school-level responses, whereas students who are referred for high levels of test anxiety can benefit from the student-level responses that would fit into Tiers 2 and 3 (targeted and intensive supports) within an MTSS model.

### 5.4. A Special Topic: Accommodations

We call out for special attention a practice that an increasing number of schools use when faced with very anxious students: testing accommodations. Formally, accommodations are provided to students with recognized disabilities. Testing accommodations change the way in which tests are administered, without changing the content itself (Lovett & Lewandowski, 2015). Anxious students often receive such accommodations, including additional time to complete tests, a separate location for tests, and clarifications of instructions for tests (Phillips et al., 2022).

In most cases, test anxiety is a poor basis for accommodations (Lovett & Nelson, 2017). Anxiety does not generally prevent a student from accessing a test, and so in most cases, test anxiety is not a disability condition. (It is, again, an extremely common experience.) Worse still, some accommodations actually encourage the avoidance that is maintaining anxiety over the long term (Phillips et al., 2022). Used poorly, accommodations send the message that the student is indeed unable to do things and thus confirm students’ irrational, negative self-beliefs. If accommodations are going to be used for anxiety, they should be faded at the earliest possible time and should be chosen to promote the student participating in assessments rather than focused on reducing distress per se.

Unfortunately, high-achieving schools may be particularly vulnerable to providing improper accommodations. Some scholars (e.g., Calarco, 2014) have argued that the same family dynamics discussed earlier can lead families to seek any services that might boost their children’s test scores. This may be why accommodations are more common in more affluent areas (Goldstein & Patel, 2019), even though poverty is a risk factor for disability. To be sure, we always assume that parents are being honest when they report that their children “need” accommodations, but parents have no reason to follow the strict legal standards that define what is “needed.” Therefore, schools must use careful, consistent decision practices to avoid simply providing whatever accommodations are being sought and thereby reinforcing the avoidance that is actually exacerbating the student’s underlying anxiety.

## 6. Conclusions

High-achieving schools have a number of features that place their students at higher risk for mental health issues, including severe forms of test anxiety. However, empirical research has greatly increased our understanding of how test anxiety works, while also providing a set of evidence-based strategies for combating it. Importantly, high-achieving schools often have more access to resources that can be used to promote these strategies. We encourage high-achieving schools to leverage these resources and begin to address test anxiety in a way consistent with scientific research on the topic, and thereby teach students a larger lesson about the perils of avoidance.

## Data Availability

Not applicable; the paper is not an empirical study.

## Abbreviations

The following abbreviations are used in this manuscript:

ISEE	Independent School Entrance Exam
SHSAT	Specialized High Schools Admissions Test
